# Toward Realization of 2.4 GHz Balunless Narrowband Receiver Front-End for Short Range Wireless Applications

**DOI:** 10.3390/s150510791

**Published:** 2015-05-07

**Authors:** Munir M. El-Desouki, Syed Manzoor Qasim, Mohammed S. BenSaleh, M. Jamal Deen

**Affiliations:** 1 King Abdulaziz City for Science and Technology (KACST), Riyadh 11442, Saudi Arabia; E-Mails: mqasim@kacst.edu.sa (S.M.Q.); mbensaleh@kacst.edu.sa (M.S.B.); 2 Department of Electrical and Computer Engineering, McMaster University, Hamilton, ON L8S 4K1, Canada; E-Mail: jamal@mcmaster.ca

**Keywords:** CMOS, narrowband receiver, radio frequency (RF), short-range, wireless sensor network (WSN)

## Abstract

The demand for radio frequency (RF) transceivers operating at 2.4 GHz band has attracted considerable research interest due to the advancement in short range wireless technologies. The performance of RF transceivers depends heavily on the transmitter and receiver front-ends. The receiver front-end is comprised of a low-noise amplifier (LNA) and a downconversion mixer. There are very few designs that focus on connecting the single-ended output LNA to a double-balanced mixer without the use of on-chip transformer, also known as a balun. The objective of designing such a receiver front-end is to achieve high integration and low power consumption. To meet these requirements, we present the design of fully-integrated 2.4 GHz receiver front-end, consisting of a narrow-band LNA and a double balanced mixer without using a balun. Here, the single-ended RF output signal of the LNA is translated into differential signal using an NMOS-PMOS (n-channel metal-oxide-semiconductor, p-channel metal-oxide-semiconductor) transistor differential pair instead of the conventional NMOS-NMOS transistor configuration, for the RF amplification stage of the double-balanced mixer. The proposed receiver circuit fabricated using TSMC 0.18 µm CMOS technology operates at 2.4 GHz and produces an output signal at 300 MHz. The fabricated receiver achieves a gain of 16.3 dB and consumes only 6.74 mW operating at 1.5 V, while utilizing 2.08 mm^2^ of chip area. Measurement results demonstrate the effectiveness and suitability of the proposed receiver for short-range wireless applications, such as in wireless sensor network (WSN).

## 1. Introduction

The demand for short-range wireless transceivers operating in the 2.4 GHz ISM (Industrial, Scientific and Medical) band, widely used in emerging ultra low-power applications, such as autonomous wireless sensor networks (WSNs) for different monitoring applications [1,2,3], Internet-of-Things (IoT) applications, and wireless body area networks (WBANs) for health care applications [4,5], has led to extensive research on the relevant system architecture and single-chip ultra low power integrated circuit designs of radio frequency (RF) transceivers [6,7,8]. The RF transceiver is a critical block in a wireless system as it consumes more than 80% of the total power of the system. In order to reduce power, the RF transceiver needs to be designed efficiently. The receiver RF front-end of a transceiver is of particular interest to many researchers as it is a critical block that consumes a significant fraction of the power. Currently, research efforts are targeted toward developing highly-integrated, low-cost and low-power single-chip RF receiver front-ends. Many of the transceivers used for short-range wireless applications utilize 2.4 GHz narrowband receivers for the RF receiver front-end designs [9,10,11].

**Figure 1:**
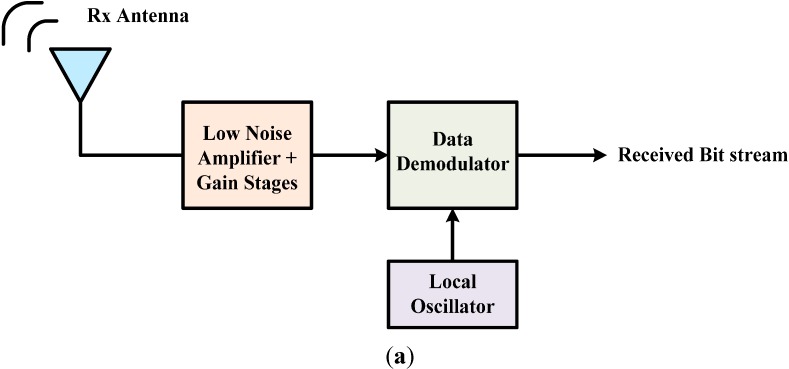

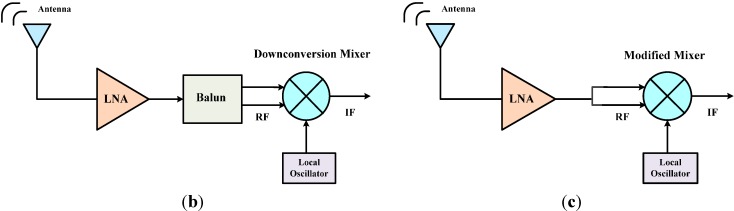
(**a**) Block diagram of a simple narrowband receiver; (**b**) Receiver front-end with LNA, balun and mixer; (**c**) Proposed balunless receiver front-end.

The block diagram of a simple narrowband receiver is shown in Figure 1a. It is comprised of a receiving antenna, low noise amplifier (LNA) with some gain stages, a data demodulator, and a local oscillator (LO). The LNA and mixer are the most power-consuming blocks of the receiver front-end [12,13]. The performance of the narrowband receiver depends mainly on the LNA and mixer. A single or double-balanced differential circuit is preferably used for mixer design, based on the gain and noise figure (NF) requirements of the system. An on-chip transformer (balun) is used to convert the single-ended or unbalanced LNA signal into differential or balanced signal for the downconversion mixer as illustrated in Figure 1b. However, integrating a balun on the chip is not only difficult but also degrades the performance of the receiver, as discussed below.

There are many published 2.4 GHz narrow-band receiver front-end designs focusing on different aspects depending on the requirements of the application. However, the problem of connecting a narrowband LNA to a double balanced mixer in a receiver front-end has not been properly addressed in the open literature and still remains a hot topic of research. There are two possible approaches to solve this problem. One solution is to use a differential LNA, which is designed to produce a differential output signal pair [14,15]. The advantage of this design is that it produces a differential output pair, which can be directly used by the double balanced mixer. It is relatively simple to fabricate and it exhibits good performance. However, it requires a differential RF input signal instead of a single input, which is problematic for practical application, since an RF signal is normally a single input signal. Additionally, it requires an additional stage in the LNA design since the two symmetric stages can produce two out-of-phase signals (the differential output pair). Therefore, the differential narrowband LNA doubles the number of stages as compared to the conventional LNA, which consumes twice the power and degrades the performance of the narrowband receiver.

Another possible solution for implementing a single input narrowband LNA followed by a double balanced mixer is to use an on-chip transformer [16]. However, this solution is also not effective, since many components need to be integrated in order to implement an on-chip transformer, which will result in an increase in chip area. This also results in greater power dissipation and increases the physical complexity of the circuit design and fabrication.

A narrowband receiver design integrating a single RF input narrowband LNA to a single balanced mixer is presented in [17]. The single balanced mixer requires a single RF input, thus eliminating the problems discussed previously. However, the single balanced mixer has high RF and LO feed-through and also the gain is only half as compared to a double balanced mixer. These drawbacks restrict this method and as a result, it is not an optimal solution for narrowband receivers.

In this paper, the design, realization, and performance evaluation of fully-integrated, low-power 2.4 GHz narrowband receiver front-end implemented in a standard 0.18 μm CMOS technology is presented. The novelty of the work is that the receiver is implemented without using an on-chip transformer to connect the LNA to double balanced mixer, as shown in Figure 1c. Specifically, the novelty of the work presented in this paper is to use an NMOS-PMOS (n-channel metal-oxide-semiconductor, p-channel metal-oxide-semiconductor) transistor differential pair, instead of the conventional NMOS-NMOS transistors [18], for the RF amplification stage of the double balanced mixer. This design achieves the single-ended LNA to double balanced mixer connection without the need for an on-chip transformer, while at the same time keeping the performance characteristics at competitive levels. The purpose of this paper is to investigate the feasibility of such a challenging design, since the mobility of both NMOS and PMOS transistors are different, making it difficult to balance the mixer.

The rest of the paper is organized as follows. In Section 2, the architecture of the narrowband receiver front-end is presented. The LNA and mixer designs are also described in detail in this section. The simulation and measurement results are discussed in detail in Section 3. The performance evaluation of the proposed receiver front-end is presented in Section 4. Finally, in Section 5, the conclusions are given.

## 2. Narrowband Receiver Front-End Architecture

**Figure 2:**
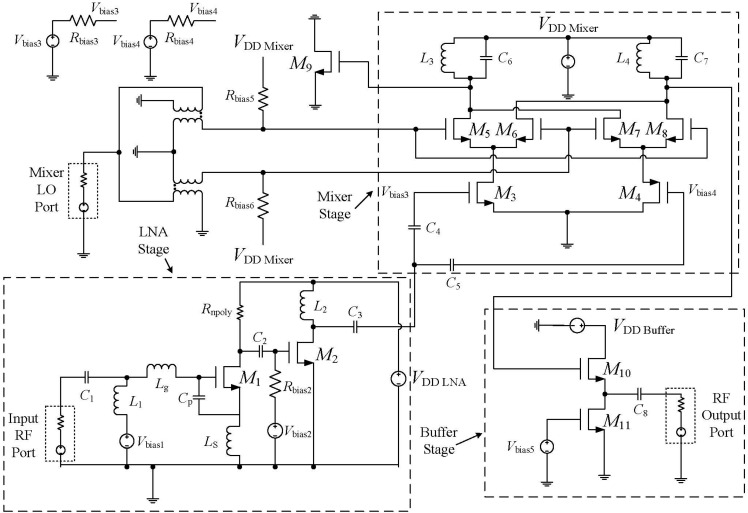
Schematic of the receiver front-end.

The narrowband receiver front-end comprises of LNA and a down-conversion double balanced mixer as shown in Figure 2, where an output buffer is added for measurements. In this work, an NMOS-PMOS differential pair was used instead of the conventional NMOS-NMOS transistors for the RF amplification stage of the double balanced mixer, so that the single RF input signal from the LNA can be translated into two differential inputs for the mixer.

### 2.1. Narrowband LNA 

**Figure 3:**
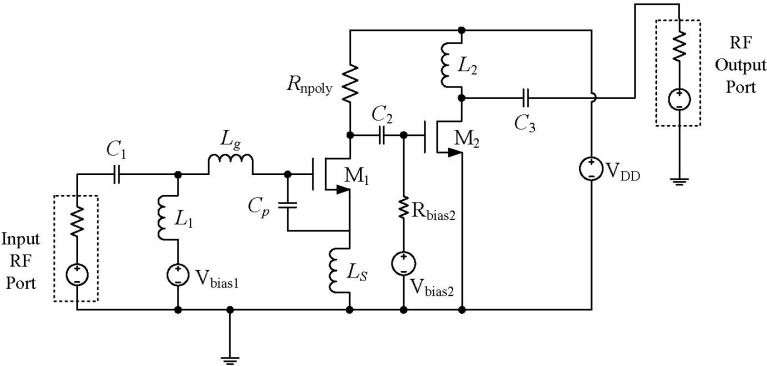
Schematic of the inductively source degenerated narrowband LNA.

LNA is one of the essential building blocks of RF receivers. In this paper, the narrowband LNA is designed as an inductively source degenerated cascade structure as shown in Figure 3. The RF signal is provided by the input RF port while the bias voltage is provided by the voltage source (*V_bias_*_1_). The capacitor *C*_1_ and inductor *L*_1_ are used to block the DC and AC signals respectively. The elements *L_g_*, *L_s_*, *C_p_* and the NMOS transistor *M*_1_ form the input matching network of the LNA [19]. The input matching impedance of this amplifier can be expressed as [20]:

(1) $$Z_{\text{in}}=\frac{1}{s(C_{gs}+C_{p})}+s(L_{s}+L_{g})+\frac{g_{m}L_{s}}{C_{gs}+C_{p}}$$

where, *g_m_* is the transconductance and *C_gs_* is the gate-source capacitance of the transistor *M*_1_.

Setting the real part of Equation (1) to be equal to the assumed source resistance of 50 Ω results in:

(2) $$\frac{g_{m}L_{s}}{C_{gs}+C_{p}}=Z_{S}=50$$

where the input matching is realized for *f* = 2.4 GHz. 

### 2.2. Downconversion Double Balanced Mixer 

**Figure 4:**
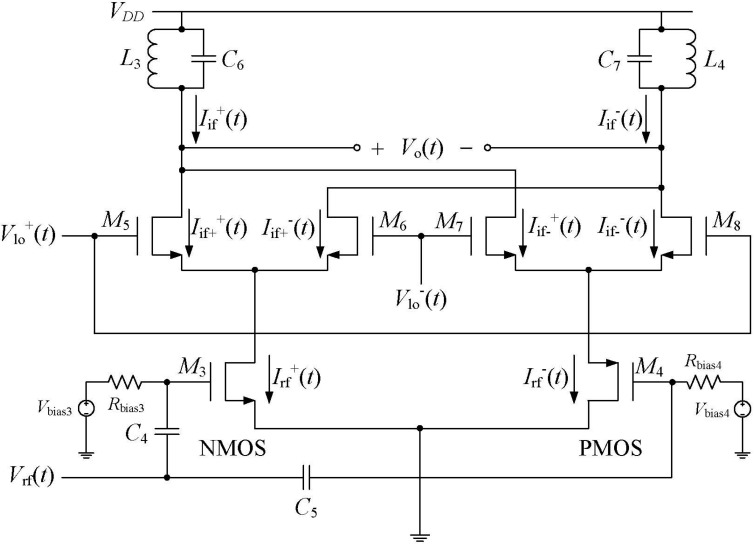
Schematic of the modified double balanced mixer.

A novel design idea is utilized so that a single RF input signal can be translated into two differential signals in the mixer, as shown in Figure 4. This modified double balanced mixer requires only a single RF output signal from the LNA, thus avoiding the use of a balun. 

The importance of this NMOS-PMOS pair is that it can create a differential current in two branches of the circuit. A simple expression for current of an NMOS transistor in the saturation region is:

(3) $$I_{D}=\frac{1}{2}\mu_{n}C_{ox}\frac{W}{L}{\left(V_{GS}-V_{t}\right)}^{2}$$

While the current of a PMOS transistor in the saturation region is:

(4) $$I_{D}=\frac{1}{2}\mu_{p}C_{ox}\frac{W}{L}{\left(V_{GS}-V_{t}\right)}^{2}$$

where, µ*_n_* and µ*_p_* are the mobilities of NMOS and PMOS transistor respectively, *W* and *L* are the width and length of the transistors respectivley, *V_GS_* and *V_t_* are the gate-source and threshold voltages, respectively.

When *V_GS_* is increasing, (*V_GS_* − *V_t_*)^2^ is increasing, which in turn means that the current *I_D_* is also increasing. On the other hand, when *V_GS_* is decreasing, the current *I_D_* is decreasing. These patterns work for both the NMOS and PMOS devices. However, from Figure 4, when *V_rf_* increases, the voltage *V_GS_* of NMOS increases while *V_GS_* of PMOS (*M*_4_) decreases, causing *I_D_* of NMOS to increase while *I_D_* of PMOS decreases and *vice versa*. The overall *V*–*I* conversion can be summarized as follows:

- When the RF input signal is increasing, the current in the NMOS branch is increasing while the current in the PMOS branch is decreasing,
- When the RF input signal is decreasing, the current in the NMOS branch is decreasing while the current in the PMOS branch is increasing.

Therefore, these two opposite currents create a differential signal pair and thus translate the single-ended RF input signal into a differential signal pair.

## 3. Simulation and Measurement Results

**Figure 5:**
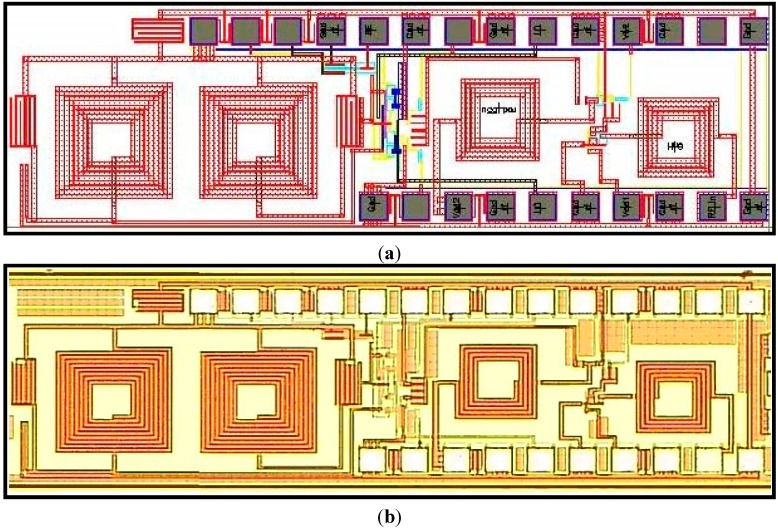
(**a**) Layout; (**b**) Die micrograph of receiver front-end.

The proposed receiver front-end is fabricated using TSMC 0.18 μm CMOS Technology as shown in Figure 5. The narrowband LNA is implemented using an inductively source degenerated cascade structure and the downconversion mixer is implemented according to double balanced configuration with complementary PMOS and NMOS transistors for the RF signal (path). The total chip area is 2.7 × 0.77 = 2.08 mm^2^.

The simulations were run using Cadence Spectre RF simulator package. A 0.18 μm CMOS process was used with device models provided by CMC Microsystems. The simulator includes the narrowband LNA, as well as the double balanced mixer. The RF input frequency is 2.4 GHz with −30 dBm power level, LO input frequency is 2.1 GHz with 0 dBm power level; IF output frequency will appear at 300 MHz and the DC supply voltage is 1.5 V.

**Figure 6:**
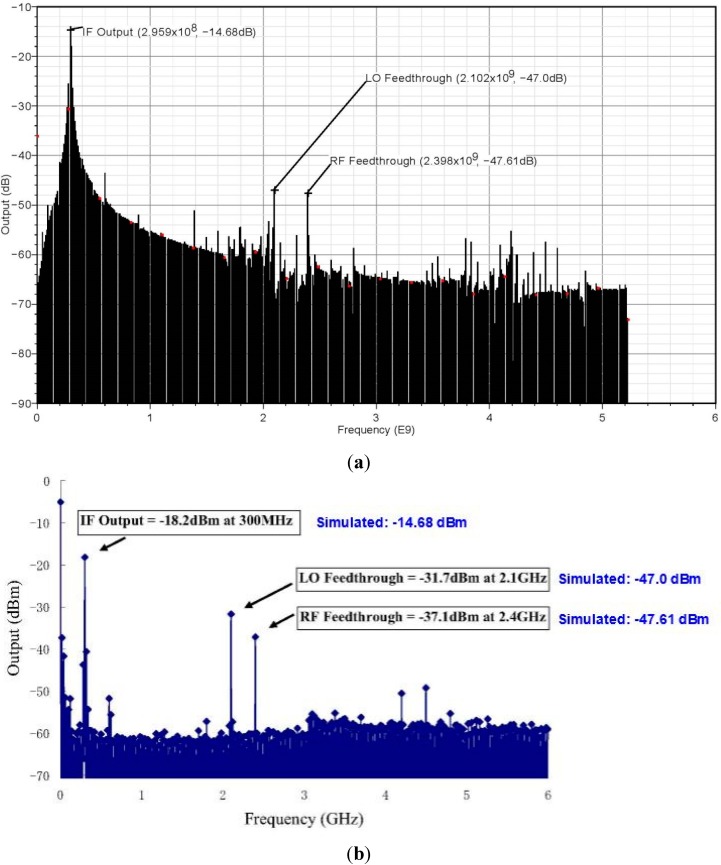
(**a**) Output of the receiver front-end; (**b**) Measured output of receiver front-end from the output data of spectrum analyzer at P_in_ = −30 dBm.

Figure 6a illustrates the simulation results for an IF output signal appearing at 300 MHz with a power amplitude of –14.68 dBm. The LO feedthrough appears at 2.1 GHz with −47.0 dBm power amplitude, and the RF feedthrough appears at 2.4 GHz with a power of −47.61 dBm. Note that the LO and RF feedthroughs have significantly lower amplitudes as compared to the desired IF output. As mentioned before, the RF input power is –30 dBm and, therefore, the gain is −14.68 − (−30) = 15.32 dBm. The measured output of the receiver front-end circuit is shown in Figure 6b. After compensating the cable losses, the measured gain of the receiver is 13.3 dB. By comparison, the simulated gain value was 15.32 dB which indicates that the measured gain was about 2 dB lower. However, the losses from the RF cables, laboratory facilities, pins or pad contacts may not have been compensated fully. This incomplete compensation of losses will affect the measured output values. Therefore, a 2 dB difference is acceptable in the measured result [21].

**Figure 7:**
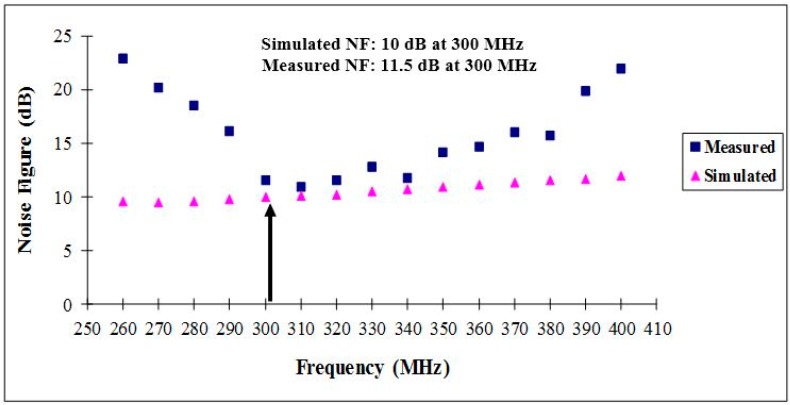
Noise figure of the receiver front-end.

Figure 7 shows the noise figure of the receiver front-end circuit. Because the IF output signal is centered at 300 MHz, this is also the frequency at which the noise figure is measured. As marked, the simulated NF is 9.99 dB at 300 MHz (the IF output frequency). The measured noise figure of the fabricated receiver circuit is 11.5 dB at 300 MHz. This indicates that the experimental noise figure is 1.5 dB higher than the simulated one which is acceptable considering the loss of 1.5 dB in the cables and that the uncertainties in the behavior of real equipment and components may affect the measurements [22,23].

**Figure 8:**
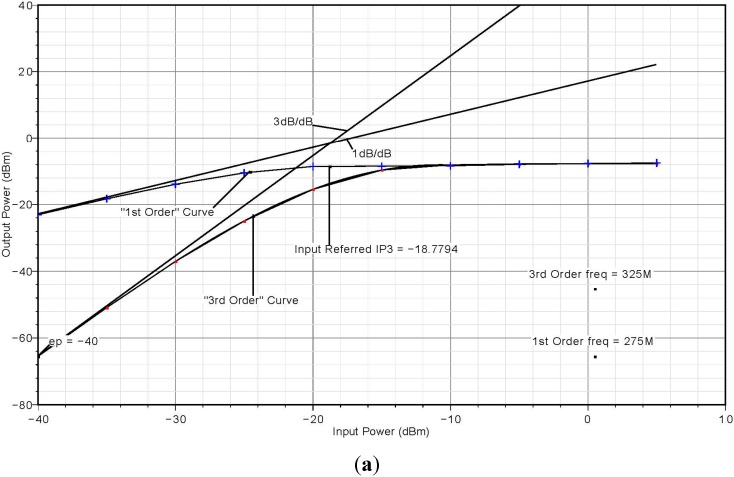

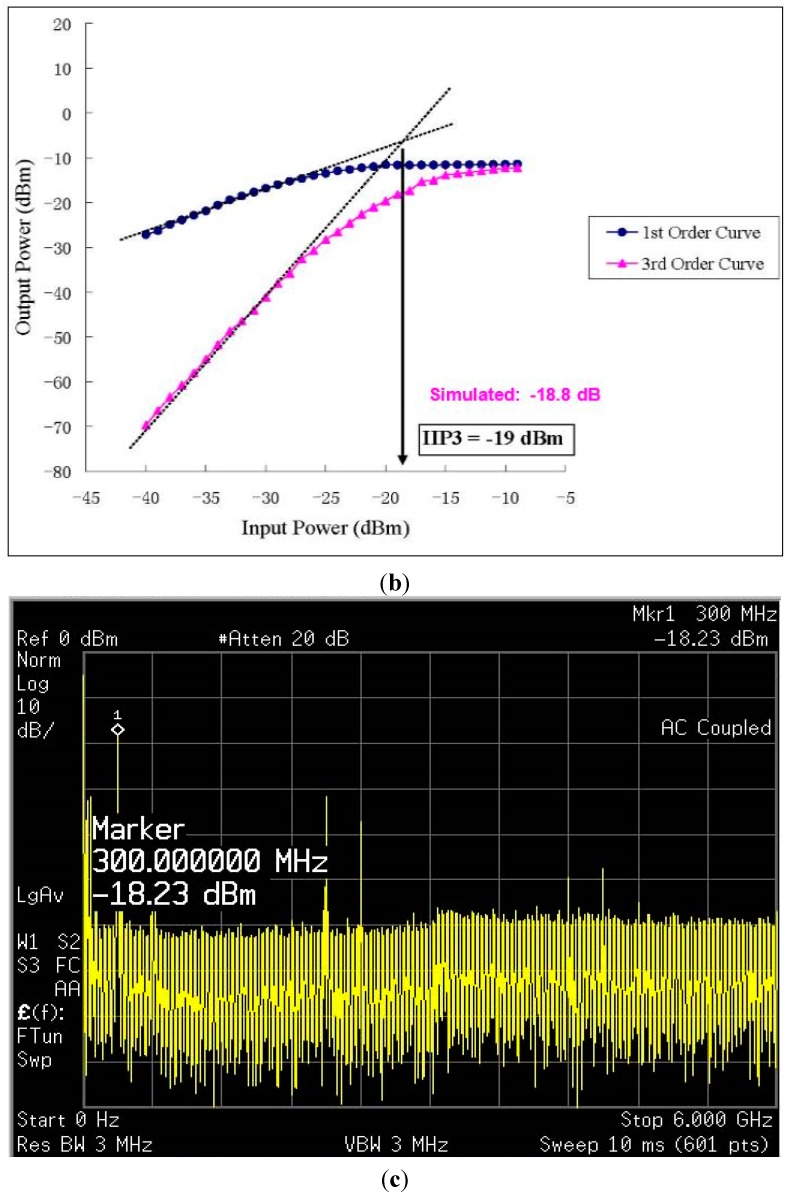
(**a**) IIP_3_ of narrowband receiver front-end; (**b**) Measured IIP_3_ of narrowband receiver front-end; (**c**) Spectrum of two-tone test.

The third-order intermodulation intercept point (IIP3) of the receiver is shown in Figure 8a–c. A *two-tone* test is applied to evaluate IIP_3_. The simulated IIP_3_ is marked and the value is found to be −18.78 dBm while the measured IIP_3_ is −19 dBm, as shown in Figure 8b. This is a reasonable result, given how close the measured value is to what was predicted by the theoretical analysis. 

The overall simulated power consumption of the receiver front-end is 5.573 mW without the output buffer and 12.035 mW with the output buffer while the measured power consumption of the receiver front-end is 6.74 mW without the output buffer and 12.85 mW with the output buffer [21].

## 4. Performance Evaluation

In order to evaluate and compare the results with previously published research, a performance metric need to be selected that accounts for all the performance parameters under discussion. Such a quantity is called a figure-of-merit (FoM), and it is defined as follows:

(5) $$\text{FoM}=20\log(f_{\text{RF}})+G+{\text{IIP}}_{3}-\text{NF}-10\log(P_{\text{diss}})$$

where, all values are measured in normalized units. The term *f*_RF_ is the frequency of the input RF signal in Hz normalized to 1 Hz, *G* is the voltage gain of the receiver in dB normalized to 1 dB, IIP_3_ is the linearity in dBm normalized to 1 dBm, NF is the noise figure in dB normalized to 1 dB, and *P*_diss_ is the power dissipation of the receiver circuit in mW normalized to 1 mW.

In Table 1, a summary of both the simulated and measured results of the proposed receiver front-end is given. In Table 2, the comparison of the designed receiver front-end with state-of-the-art designs is presented. From Table 2, it is observed that the FoM of the proposed receiver front-end, comprising of LNA and modified mixer (to avoid the use of balun), is competitive at 0.18 µm CMOS technology, with the performance comparable and acceptable for short range low voltage low power wireless applications. 

**Table 1:** Performance summary of the receiver front-end.

	Simulated	Measured
CMOS Technology (µm)	0.18	0.18
RF frequency (GHz)	2.40	2.40
Gain (Differential)	18.2	16.3
Gain (Single-ended)	15.3	13.3
NF (dB)	10.0	11.5
IIP_3_ (dBm)	**−**18.8	**−**19.0
Power dissipation (without buffer) (mW)	5.573	6.74
Power dissipation (with buffer) (mW)	12.035	12.85

**Table 2:** Performance comparison with related work.

	CMOS Tech. (µm)	Frequency (GHz)	Gain (dB)	NF (dB)	IIP3 (dBm)	P_diss_ (mW)	Chip Area (mm^2^)	FoM
**Simulated**	**0.18**	**2.4**	**18.2**	**10**	**−18.8**	**5.57**	**2.08**	**170**
**Measured**	**0.18**	**2.4**	**16.3**	**11.5**	**−19**	**6.74**	**2.08**	**165**
**[24]**	0.09	2.4	30	18	−22	8.5	3.4	168
**[25]**	0.13	2.4	12.5	28	−21	3.4	3.3	146
**[26]**	0.18	2.44	8	8.9	−13.5	2.754	**^††^**	169
**[27]**	0.25	2.4	14.5	4.8	−18	11.3	1.82	169
**[28]**	0.18	2.4	21.4	13.9	−18	6.5	**NA**	169
**[29]**	0.18	2.4	20.4	19	**NA**	0.5	0.765 **	-
**[30]**	0.13	2.1	15 *	15.7 *	−3 *	12	1.1	172
**[31]**	0.13	2.4	12 *	30 *	0 *	25.2	0.612 **	156
**[32]**	0.18	2.4	18	8	−15	8.7	0.263 **	173

^††^ Only Simulation results, chip was not fabricated. NA: Not available. * Measurement results for low gain, ** Active chip area.

Because the standard RF input of a mixer is single-ended, the receiver front-end usually requires either a pair of differential inputs to the LNA, or an on-chip transformer to create the inputs for the double balanced mixer. However, an on-chip transformer is relatively hard to realize, requires more chip area, and consumes more power due to its low on-chip quality factor, resulting also in degraded circuit performance. Therefore, the benefit of the proposed receiver design is that it requires only a single RF input signal to provide an output IF (intermediate frequency) signal. An on-chip transformer or Balun is not required, which means that the single RF output signal can be translated into a differential signal pair without adding any elements in the circuit. Overall, the proposed design exhibits the following advantages:

- requires a single-ended RF input without the need of using on-chip transformer/balun;
- has a simple circuit architecture that eases the process of fabrication;
- reduces power consumption by avoiding on-chip lossy reactive elements;
- provides competitive overall performance compared to other designs.

However, there are tradeoffs involved with this receiver design. Since a single-ended RF signal is converted into a set of differential signals using a PMOS-NMOS transistor pair instead of an NMOS differential pair, there are challenges in balancing the currents within the mixer due to the different mobilities of the PMOS and NMOS transistors. Therefore, the PMOS transistor usually requires a larger size to provide the same peak current as the NMOS device. However, this also poses challenges in terms of matching the phases in both branches since the larger transistor will operate slower than the smaller one due to its larger capacitances. In this work, manual offset bias calibration was used to balance both branches during measurements. As part of the future work, the use of an automated feedback loop will be investigated to balance the branches on-chip with minimal user intervention. 

## 5. Conclusions

In this paper, design and CMOS realization of 2.4 GHz narrowband receiver front-end for short-range low-voltage low-power wireless applications is presented. A significant problem with narrowband receiver is that the amplifier requires either a differential input, or an on-chip transformer (balun) to translate the single-ended RF output signal of the LNA into a differential signal pair as the input of the double balanced mixer. This paper mainly contributes toward solving the problem of connecting a LNA to a double-balanced mixer. The new double-balanced mixer needs only a single RF input from the LNA, and does not require a balun for the single-to-differential conversion. Fabricated using a TSMC 0.18 µm CMOS process through CMC, the proposed receiver achieves a voltage gain of 16.3 dB, a noise figure of 11.5 dB, and IIP3 of −19 dBm. Measurement results of the 2.4 GHz narrowband receiver front-end demonstrate that it can achieve competitive performance levels, while reducing receiver chip area and power consumption, resulting in simpler and efficient designs of RF transceivers that are suitable for emerging low power applications, such as WSN.
